# Pesticides in the Indoor Environment of Residential Houses: A Case Study in Strasbourg, France

**DOI:** 10.3390/ijerph192114049

**Published:** 2022-10-28

**Authors:** Josephine Al-Alam, Alexandre Sonnette, Olivier Delhomme, Laurent Y. Alleman, Patrice Coddeville, Maurice Millet

**Affiliations:** 1 Civil Engineering Department, Lebanese American University, 309 Bassil Building, Byblos 1102, Lebanon; 2 Institut de Chimie et Procédés pour l’Energie, l’Environnement et la Santé (ICPEES-UMR 7515 CNRS), Université de Strasbourg, Equipe de Physico-Chimie de l’Atmosphère, F-67087 Strasbourg, France; 3 LTSER France, Zone Atelier Environnementale Urbaine, Maison Interuniversitaire des Sciences de l’Homme-Alsace (MISHA), 5, Allée Du Général Rouvillois, CS 50008, F-67083 Strasbourg, France; 4 IMT Nord Europe, Institut Mines-Télécom, University Lille, Centre for Energy and Environment, F-59000 Lille, France; 5 Université de Lorraine—UFR Sciences Fondamentales et Appliquées (SciFa), Campus Bridoux, F-57070 Metz, France

**Keywords:** pesticides, indoor environment, air, dust, pyrethroids

## Abstract

Indoor environmental exposure to pesticides has become one of the major concerns that might adversely affect human health and development. People spend most of their lifetime in enclosed indoor environments where they might inhale harmful toxic chemicals, such as pesticides, dispersed either in particulate or in a gas phase. In this study, an assessment of pesticide contamination in indoor environments was conducted. The study covered nine houses during one year, starting from February 2016 and ending in February 2017, in which both air and dust samples were assessed for their potential contamination with 50 pesticides. The results showed that all the assessed houses were contaminated by several pesticides, especially with the allethrin pesticide (detection frequency (DF) = 100%). The highest pesticide contamination was detected in the spring/summer season when it reached an average of around 185 ng g^−1^ and 186.4 ng sampler^−1^ in the collected dust and air samples, respectively. The potential contamination of pyrethroid insecticides within all the targeted samples revealed by this study stresses the importance of minimizing the use of such indoor treatments as part of the efficient prevention and control of human exposure to pesticides.

## 1. Introduction

Indoor environmental contamination is a major concern because of its direct adverse health effects on humans [1]. Typically, people spend almost 90% of their time indoors, whether in their houses, dwellings, workplaces, or schools, where they can be constantly exposed to organic pollutants [2,3,4]. Accordingly, indoor environmental quality monitoring studies became a must for identifying the levels and sources of these pollutants indoors, enabling for their control [5,6]. Studies have reported that semivolatile organic compounds (SVOCs) are, in association with volatile organic compounds and particulate matter, the main source of indoor environmental-quality alteration [5,6,7]. Upon their emission, SVOCs can diffuse in a gas phase, particulate phase, and within settled dust, leading to chronic human exposure [8].

Polycyclic aromatic hydrocarbons (PAHs), polychlorinated biphenyls (PCBs), phthalates, and pesticides are among the major SVOCs present in indoor environments [3]. In fact, despite being mainly used in agricultural activities, pesticides can be widely used indoors for domestic activities, such as for pest control [6,9,10]. In addition, pesticides can infiltrate the indoor environment through dispersion following their field application [11]. Indeed, it was shown that pesticides might persist at higher rates in closed indoor environments as compared to outdoor environments due to the lack of thermal, oxidative (O_3_), and microbial activities [12]. As a result, people are more prone to pesticide exposure in indoor environments, where pyrethroid and organophosphate pesticides are found to be intensively used [5,13,14]. The indoor exposure to pesticides is mainly associated with severe health problems, including irritation, lower respiratory pain, dyspnea, dry cough, pediatric brain tumors, birth defects, childhood leukemia, developmental inhibition, and motor skill reduction, as well as severe damage to the liver, kidneys, and the endocrine and nervous systems [15,16].

Depending on their physicochemical properties, such as vapor pressure, K_OW_, viscosity, and solubility, pesticides can be found in the air, either in particulate or in a gas phase [17]. Air and dust are known to be very relevant matrices for the assessment of pesticide exposure indoors [18]. However, such assessments of indoor environments require a simultaneous assessment of air and dust contamination as, to date, no direct link between these SVOCs in the air and in dust has been established. Moreover, it was shown that SVOC equilibrium concentrations are commonly higher in dust particles than in the gaseous portion of indoor air [19].

Accordingly, the aim of this study, conducted over one year, was to assess pesticide contamination of indoor environmental quality in nine different residences in the Strasbourg area using both air and dust particles. Air sampling was carried out using Tenax^®^-TA adsorbing cartridges as passive samplers, while dust sampling was conducted using a vacuum cleaner fitted with SiC^©^ foams. To the best of our knowledge, the use of these two techniques for air and dust monitoring has not previously been reported (for the simultaneous monitoring of pesticides in indoor environments).

## 2. Materials and Methods

### 2.1. Sampling Sites

Nine residential houses were monitored in this study. The targeted houses were all situated in the Alsace region and followed the suggestions of the Association for the Prevention of Atmospheric Pollution (APPA) and the Association for the Monitoring and the study of Air Pollution in Alsace (ASPA). In fact, the chosen houses were selected based on the recommendations of the ASPA, mainly based on the request of the residents themselves to study their houses. As a result, the main selection criterion was the acceptance of the residents to participate in the study, which was carried out over 1 year inside the houses. In addition, the nine houses were found to be representative of different geographical locations and, therefore, seemed to be of interest for the main goal of the study, which is the assessment of the overall indoor air quality in the Strasbourg region. Sampling was conducted for one year, starting from February 2016 and ending in February 2017.

**Figure 1:**
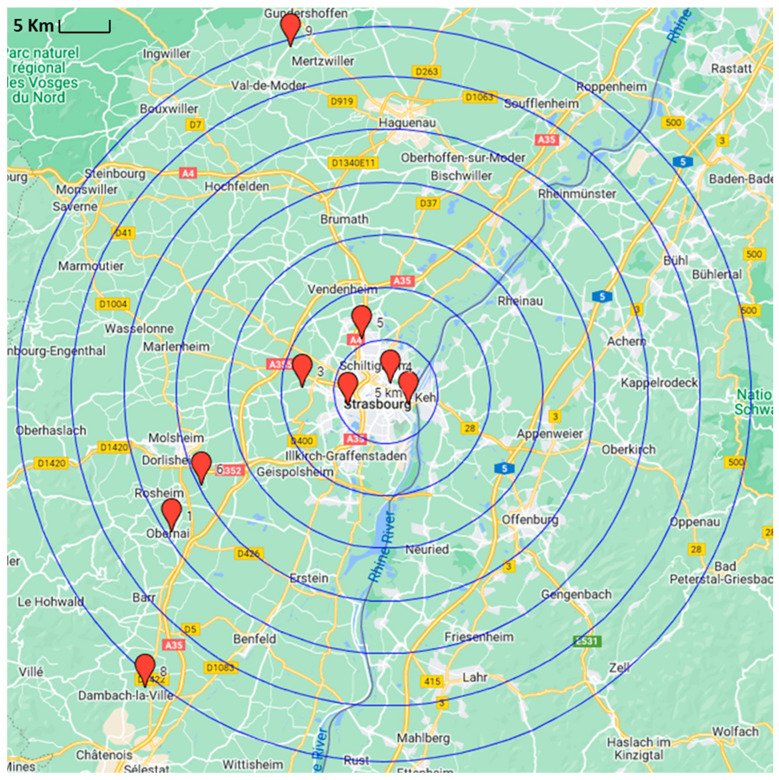
Geographical distribution of the targeted sites (houses are represented by red pins).

Air samples were collected from the living rooms and the bedrooms of all the targeted houses to check the influence of the room on pesticide accumulation by the sampler. The geographical distributions of the targeted sites are shown in Figure 1. The interactive map pertaining to each sampling location, in addition to a recapitulative table summarizing relevant information on each sampling site, is shown in Supplementary Material S1.

### 2.2. Sampling Campaigns

Sampling was carried out monthly for one year, during which both air and dust were monitored in each residence.

For air monitoring, Radiello^®^ Tenax-TA^®^ tubes, purchased from Sigma Aldrich, L’Isle d’Abeau (France), were coated in plastic shelters, according to the model developed by Wania et al., in 2003 [20]. These shelters aimed to protect the sampler tubes from indoor wind variations. The Tenax tubes were exposed to the indoor air of the targeted rooms for 15 days, and their accumulations were reported as a monthly average (average of 2 samples/month) so that the data would be coherent with the dust sampling data conducted monthly. Directly after exposure to the pollutants, the Radiello^®^ Tenax^®^-TA adsorbing cartridges were transferred into their original capped glass tubes, where they were stored at −18 °C until further analysis.

To ascertain the potential contribution of the dust-borne pesticides to indoor air contamination, the dust samples were sampled on a 2 m^2^ surface area in the nine different residences. The concentration of the collected dust samples varied between 0.05 and 0.3 g, with an average of 0.1 g. For dust monitoring, SiC^©^ foams were fitted in the sampling head of a custom-made vacuum cleaner based on the model developed by Sonnette et al. in 2021 [21]. Dust sampling was carried out on a 2 m^2^ surface area, after which the foams were directly wrapped in aluminum foils and stored at −18 °C until their extraction and further analysis.

#### Passive Samplers

For air quality monitoring, Radiello^®^ Tenax^®^-TA adsorbing cartridges (100 mesh, 4.8 mm diameter) were used. Prior to their field deployment, Tenax^®^-TA adsorbing cartridges were subjected to conditioning for 45 min at 350 °C using Helium (99.99%) at 45 mL min^−1^. Afterward, the conditioned cartridges were stored in capped glass tubes enclosed with Teflon at room temperature until exposure.

For dust monitoring, the SiC^©^ foams were used as adsorbents. These foams were extruded in the sampling head of a vacuum cleaner. Prior to their use, the SiC^©^ foams were subjected to PSE cleaning, according to the procedure used by Al-Alam et al., 2020 [22]. Afterward, the treated foams were wrapped in aluminum foils, weighed, stored for two weeks at 50 °C, and then assessed for their potential contamination with the targeted pesticides.

### 2.3. Analytical Procedure

#### 2.3.1. Reagents and Chemicals

Acetonitrile (ACN), *n*-hexane, and toluene of HPLC quality were purchased from Sigma-Aldrich (St. Quentin Fallavier, France).

Standards of 50 individual pesticides of Pestanal^®^ quality (>99% purity) and *N*-*tert*-butyldimethylsilyl-***N***-methyltrifluoroacetamide (M***t***BSTFA) were purchased from Fluka (Sigma Aldrich, St. Quentin Fallavier, France), Dr Ehrenstorfer GmbH (Cluzeau Info Labo, Sainte-Foy-la-Grande, France), or Riedel de Haën (Sigma Aldrich, St. Quentin Fallavier, France). A mixture of these pesticides (SM1) was prepared at 10 mg L^–1^ in ACN and stored at −18 °C. The list of pesticides under study is summarized in Supplementary Material S2.

Five internal standards (IS) were used, including trifluralin d_14_, nitrophenol d_4_, *pp’*-DDE d_8_, *pp’*-DDT d_8_, sand trans-cypermethrin d_6_. These ISs were obtained from Sigma-Aldrich (L’Isle d’Abeau, France) and Cambridge isotope laboratories (Cluzeau Info Labo, Sainte-Foy-la-Grande, France). A mixture of these ISs at 10 mg L^−1^ in ACN were prepared and used for the quantification of the 50 assessed pesticides.

#### 2.3.2. Pesticides Extraction from Dust Samples

After their field exposure, the SiC^©^ foams were extracted using a pressurized solvent extraction method (PSE) based on a modification of the method developed by Schummer et al. in 2012 [23]. Accordingly, the foams were introduced to the 33 mL stainless steel extraction cells, where they were subjected to two 10 min PSE cycles using ACN (100%) and were then extracted at 150 °C and a pressure of 1500 psi. Afterward, a flow of high-purity nitrogen was applied for 5 min, and the collected extracts were concentrated under a fume hood, then reconstituted to 1 mL using ACN.

#### 2.3.3. Pesticides Analysis from Air and Dust Samples

Pesticides collected from both the air and dust samples were analyzed using ATD-GC/MSMS, as previously developed by Sonnette et al. in 2021 [21].

An ATD 350 was coupled to a GC–MS/MS system through a valve operating at 280 °C, and a transfer line operated at 300 °C. Helium (He), at 45 mL min^−1^, was used for the two thermal desorption steps. Collected compounds were then analyzed on a Macherey-Nagel OPTIMA XLB capillary column (60 m × 0.25 mm; 0.25 µm), with He as the carrier gas operating at 1.2 mL min^−1^. The ion source was heated at 200 °C, while the transfer line was kept at 300 °C. The oven ramp was as follows: 50 °C for 3 min, followed by an increase in the temperature to 240 °C with a heating rate of 40 °C min, followed by additional heating to 255 °C at a rate of 1.5 °C/min, where it remained constant for 5 min. Finally, the temperature was increased at a rate of 20 °C/min to reach 330 °C, where it was held for 18 min. Details of the MRM method are summarized in Supplementary Material S3.

### 2.4. QA/QC

For the QA/QC analysis, cleaned matrices were used as adsorbent blanks. After being cleaned, each of the assessed adsorbents were spiked with 100 μL of a mixture of the tested pesticides at 10 mg L^−1^, prepared in ACN. These spiked samplers undergo the same extraction and analytical procedure used for real sample analysis (developed in the analytical procedure section. The analysis of these spiked samplers showed no significant difference to a pure standard mixture at the same level of concentration to the spiked pollutants, proving, therefore, the complete absence of the targeted pollutants in the nonspiked blank matrices. Method validation parameters, including details of the limits of detection and quantification and uncertainties are shown in Supplementary Material S4.

For a comparison of the average mean pesticide concentrations, a paired *t*-Test analysis of means (*p* = 0.05) was used.

## 3. Results

### 3.1. Pesticides in Dust Samples

**Figure 2:**
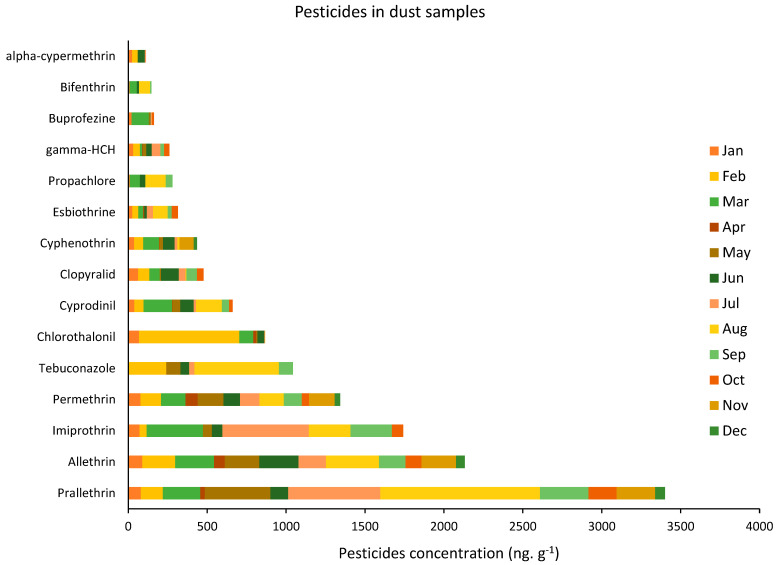
Monthly average concentrations of 15 pesticides detected in dust samples in the nine residences from 2016−2017 (data are shown as per average concentrations of pesticides detected in the nine residences per month).

Among the 50 assessed pesticides, only 15 pesticides were regularly detected in almost all of the nine targeted residences. The concentrations of those 15 pesticides in the dust samples during the one-year sampling campaign are shown in Figure 2. The average concentration of the pesticides analyzed in the collected dust samples varied between 9 ng g^−1^ for alpha-cypermethrin and 283 ng g^−1^ for prallethrin, with an average total concentration of 74 ng g^−1^. Moreover, the results showed that, among the 50 targeted pesticides, the highest concentrations were retrieved for the four main pyrethroids insecticides: prallethrin, allethrin, imiprothrin, and permethrin, with average concentrations of approximately 283, 177, 145, and 112 ng g^−1^, respectively. In addition, the obtained results showed that the highest concentration of the pesticides was in August when the average pesticide concentration reached approximately 185 ng g^−1^.

### 3.2. Pesticides in Air Samples

**Figure 3:**
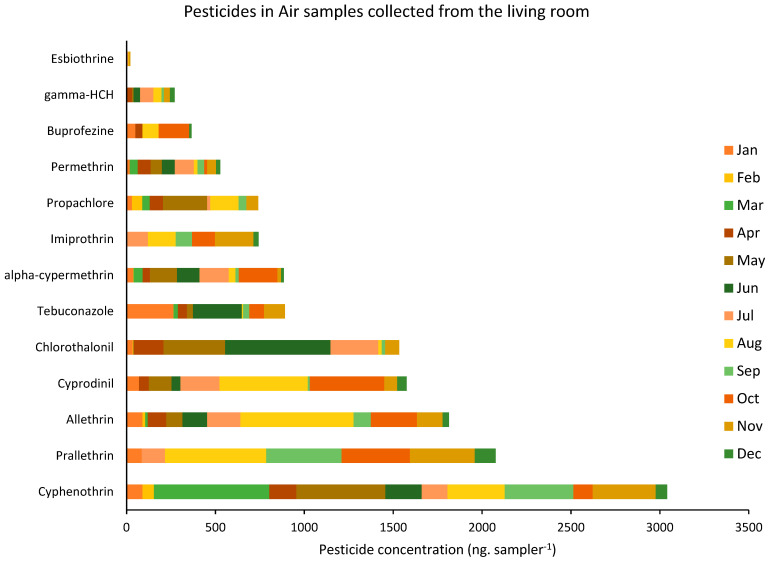
Variation of the concentration of the pesticides detected in the air samples collected from the living rooms of the targeted houses during the one-year sampling campaign (data are shown as per average concentration of pesticides detected in the nine residences per month).

**Figure 4:**
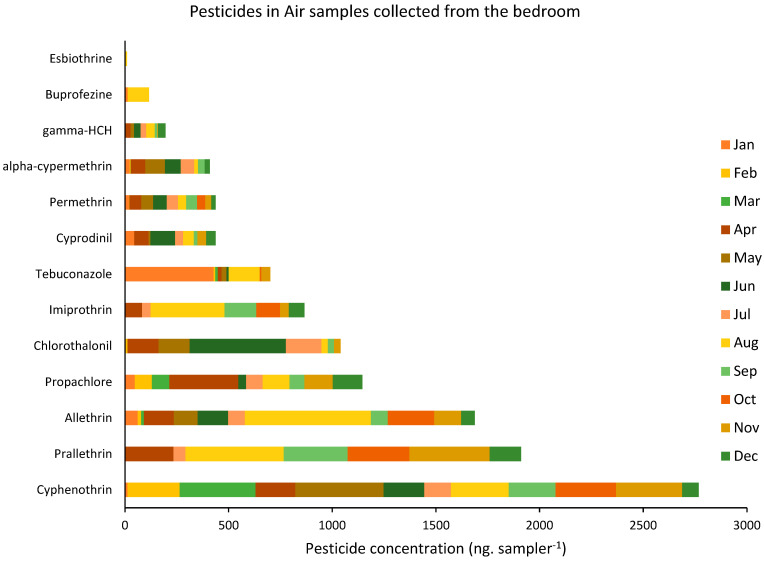
Variation of the concentration of pesticides detected in the air samples collected from the bedrooms of the targeted houses during the one-year sampling campaign (data are shown as per average concentration of pesticides detected in the nine residences per month).

Among all the assessed pesticides, 13 pesticides were regularly detected in almost all the analyzed residences. Figure 3 and Figure 4 show the concentration of those 13 pesticides, which were detected in the air samples collected, respectively, from the living room and the bedroom of all the targeted houses during the one-year sampling campaign.

**Figure 5:**
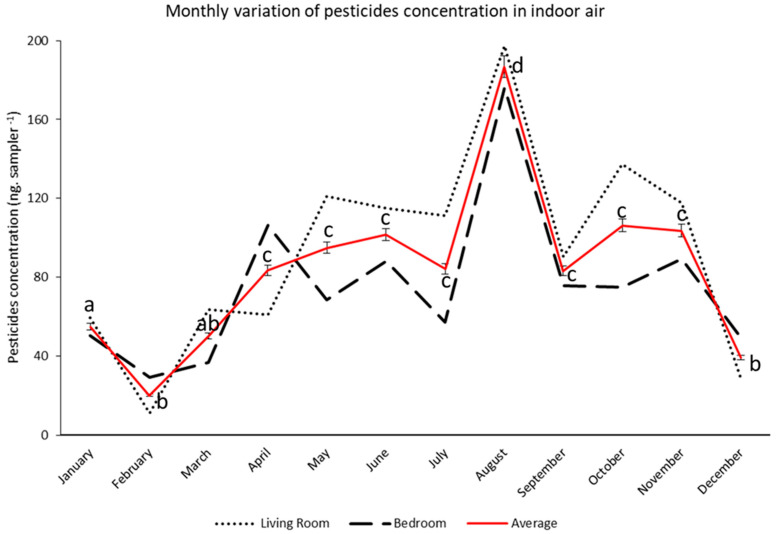
Monthly variations of the concentration of pesticides detected in the air samples collected from the living rooms and bedrooms of the nine targeted houses during the one-year sampling campaign (data are shown as per average concentration of pesticides detected in the nine residences per month). Differing lowercase denotes the means of averages that are significantly different (*p* < 0.05) between each month.

The comparison of the monthly average concentrations of the pesticides in the air samples collected from both the living rooms and bedrooms of the targeted residences, illustrated in Figure 5, yielded no significant variations, as per the paired t-Test analysis of the means (*p* = 0.05). Accordingly, the data collected for pesticide contamination in the indoor air was treated and presented as the average concentration of those pesticides detected in both rooms.

The average concentration of pesticides analyzed in the collected air samples varied between 1.34 ng sampler^−1^ for esbiothrin and 242.05 ng sampler^−1^ for cyphenothrin, with an average total of 84 ng sampler^−1^. Similar to the dust analysis, among the 50 targeted pesticides, the highest concentrations belonged to the pyrethroids insecticides, among which cyphenothrin, prallethrin, and allethrin were the most concentrated, with average concentrations of around 242.05, 165.7, and 145.9 ng sampler^−1^, respectively. Additionally, the highest concentration of pesticides was found in August when they reached an average of 186.4 ng sampler^−1^ in the assessed air samples.

### 3.3. Pesticides in Dust and Air Samples

**Figure 6:**
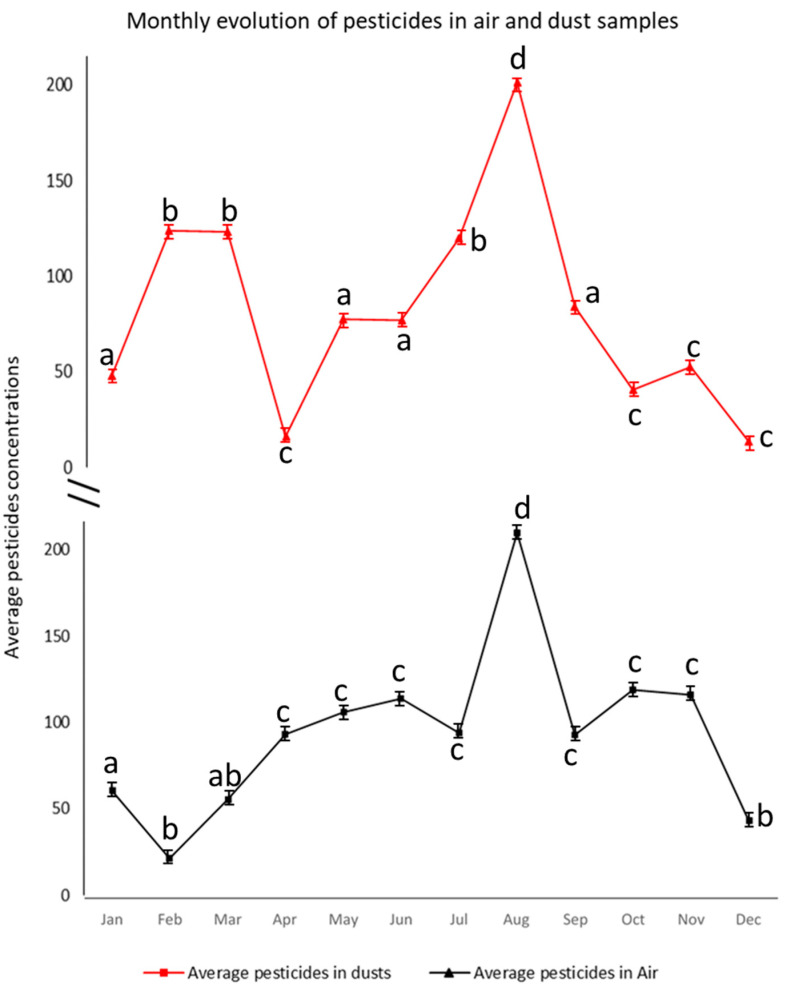
Monthly evolution of the average of the pesticides detected in the dust (ng g^−1^) and air (ng sampler^−1^) samples; differing lowercase denotes the means of averages that are significantly different (*p* < 0.05) between each month.

The comparison of air and dust matrices, illustrated in Figure 6, showed that indoor dust and airborne particles present similar trends regarding the level of pesticide contamination.

These results revealed that the highest concentration of pesticides in both indoor air and dust was achieved in August when the average concentration of the pesticides detected in the dust and air samples reached 185 ng g^−^^1^ and 186.4 ng sampler^−^^1^, respectively. The obtained results showed that the average levels of the pesticides detected in the indoor dust and air samples tended to be higher in the spring/summer season, accounting for the period between April and September (average of around 87.7 ng g^−^^1^ and 105.6 ng sampler^−^^1^, respectively), when compared to those detected in the fall/winter season (between October and March, with an average of around 60.9 ng g^−^^1^ and 62.3 ng sampler^−^^1^, respectively). The average seasonal variation of the pesticides in the air and dust samples is shown in Supplementary Material S5.

**Figure 7:**
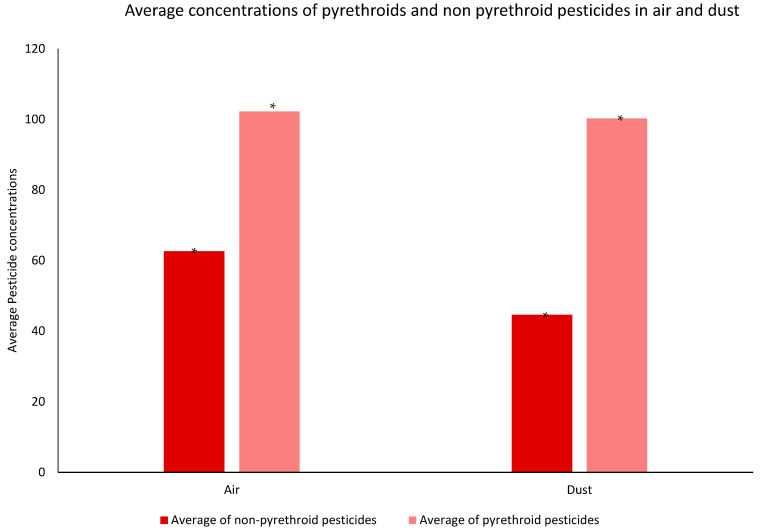
Average concentration of pyrethroid and nonpyrethroid pesticides in air (ng sampler^−1^) and dust (ng g^−1^) over one year in the nine residences assessed. Asterisks (*) denotes the means that are significantly different (*p* < 0.05) between the pyrethroids and nonpyrethroid pesticides in each matrix.

In addition, the comparison of the type of pesticides detected in each of the two tested matrices (Figure 7) showed that both the air and dust samples collected were significantly contaminated with pyrethroids insecticides (*p* < 0.05).

## 4. Discussion

The occurrence of several pesticides in the indoor air and dust samples collected from nine houses in the Alsace region revealed the wide usage and/or migration of these compounds within/into the indoor environment.

As reported by previous studies, indoor pesticide contamination could occur through the direct domestic application of the insecticides or through the infiltration of these particles from outdoor fields [11,24,25]. The average concentrations of cyprodinil in indoor air (83.9 ng sampler^−1^) and tebuconazole (66.3 ng sampler^−1^) were of the same order of magnitude as those reported by Raeppel et al. in 2016 (less than 100 ng g^−1^) [26]. The average indoor concentrations of the pesticides, calculated during the one-year monitoring campaign, were higher than those reported by previous studies for outdoor air analysis, especially during the fall/winter period, when no pesticides were detected from November to February in rural areas in Luxembourg [27]. The obtained results suggest that the occurrence of pesticides in the indoor environment throughout the year might be generated from indoor applications. However, limited air movement and reduced degradation rates (indoors) could lead to the severe accumulation of such compounds indoors, despite originating from outdoor applications. In fact, the nature of the indoor environment forms a barrier for the flux of pollutants, which emphasizes the persistence of these pollutants at higher rates when compared to outdoor environments, where they are subjected to severe degradation processes [28].

In this study, both air and dust samples were collected and were found to be significant contaminated with pyrethroids insecticides, mainly with prallethrin, allethrin, permethrin, and cyphenothrin (Figure 6). These pesticides are known as relatively volatile pyrethroids (Supplementary Material S6) with repellent properties against mosquitoes [29]. They are characterized by low vapor pressure and relatively high octanol/water partition coefficients, enabling their binding to particulate matter collected from indoor dust [30]. These findings are in accordance with those previously reported by several studies on the prevalence of these pyrethroids in indoor environments collected from 30 French residences, where pyrethrins and their synthetic analogs (pyrethroids), specifically permethrin, cypermethrin, and allethrin, were found to be the most frequently used insecticides that tend to partition into the dust and may degrade more slowly, allowing for their accumulation over time from repeated applications [31]. The significant abundance of allethrin in the collected samples is similar to the results provided by Quirós-Alcalá et al. in 2011 for the assessment of pesticides in household dust in California, in which allethrin was commonly detected in the majority of the assessed samples (detection frequency (DF) ≥ 80%) [32]. In fact, allethrin is an effective domestic insecticide used against domestic flies and mosquitoes. This pesticide is mainly used for indoor insecticide treatment. This insecticide is rarely used in agriculture due to its sensitivity to photodegradation [33].

In addition, permethrin was found to be the major pyrethroid insecticide detected in household dust samples collected from rural and urban areas in France, where it reached an average of 770 ng g^−1^ [34].

Furthermore, the results obtained in this study showed that almost all the pesticides detected in the air samples were found in the correspondent dust samples, while the reverse was not always correct. Indeed, the analysis of the 13 pesticides (in air samples collected from all the residences) regularly showed that these pesticides were among the 15 pesticides most regularly detected in the dust samples collected from all the residences. These results showed that these detected pesticides were majorly concentrated in the dust samples as compared to the air samples. For instance, in the dust samples, both allethrin and permethrin were found at a DF of 100%; in the air samples, it was found at a DF of 100–75%. In addition, clopyralid and bifenthrin were only found in the dust samples at a DF percentage of around 53 and 15%, respectively. These results are in accordance with previous studies conducted in New Jersey [32,35,36], which showed that, for semivolatile pesticides, the majority of domestic pesticides are detected at higher levels in dust samples, where they reach up to 10^6^ as high as the concentrations of pesticides found in indoor air samples.

The seasonal analysis of the pesticides in both matrices revealed higher concentration levels during the spring/summer period. These results were also proved by other studies evidencing the fact that higher levels of pesticides are found in summer when pulverized pesticides can evaporate at high temperatures, attach to air particles, and then transmit to indoor sites by diffusion [37,38]. In addition, it is worth noting that the main pesticide contamination in this study is dedicated to the pyrethroids insecticides commonly used to control various types of insects in and around residential dwellings [39], and, accordingly, they are supposed to be heavily used to control pests, especially during summer where the control of mosquitos is needed and is greater [40].

## 5. Conclusions

In conclusion, the findings of the sampling campaign conducted in this work showed that both air and dust samples could be used for indoor environmental assessment. Overall, around 13 pesticides were consistently detected in the indoor environments of the nine residences in Strasbourg and their surroundings, with higher concentrations during the spring/summer season. The obtained results showed that pyrethroids insecticides appeared to be the main source of pesticide contamination, in particular, allethrin, which was found in 100% of the assessed dwellings (in both air and dust samples). The large prevalence of this insecticide urges a need for strategic prevention to minimize an individual’s exposure to such harmful products.

The obtained results suggest that the presence of pesticides indoors is mainly due to either their direct usage for domestic treatment, especially for pyrethroids insecticides or to their infiltration from the outdoor environment, following on from their volatilization. Accordingly, further evaluations of indoor pesticide contamination need to be assessed while taking into consideration outdoor pesticide contamination, as well as larger sampling areas, in order to fully understand the source and mechanisms by which these compounds may migrate into the indoor environment. In addition, further studies aiming to field-calibrate the samplers need to be conducted in order to overcome the limitations imposed by the fluctuations occurring in field-deployment physical–chemical conditions.

## Data Availability

Data are available upon request.

## Supplementary Materials

The following supporting information can be downloaded at: https://www.mdpi.com/article/10.3390/ijerph192114049/s1, Supplementary Material S1: Interactive map of the sampling locations, Table S1: Sampling Sites’ details, Supplementary Material S2: Standards of individual pesticides used, Supplementary Material S3: Retention time (RT), precursor ion and product ions with associated dissociation energy value in GC-MSMS for each pesticide, Supplementary Material S4: ATD-GC/MSMS method performance and validation for pesticides analysis, Supplementary Material S5: Seasonal Variation of pesticides in air and dust samples, Supplementary Material S6: Classification of detected pesticides.
