# Supplementary material for: Pesticides in the Indoor Environment of Residential Houses: A Case Study in Strasbourg, France
Source: Int J Environ Res Public Health. 2022 Oct 28;19(21):14049. doi: 10.3390/ijerph192114049 (PMC9658446; doi:10.3390/ijerph192114049)
Supplement: Supplementary file 1 [file ijerph-19-14049-s001.zip › ijerph-1938710-supplementary.pdf]

Supplementary Material S1:

Interactive map of the sampling locations: https://www.google.com/maps/d/edit?mid=1jar58UraZyclgQno7qF7_fFbSYGQ7hs&usp=sharing,

Table S1: Sampling Sites' details

Reference	ZIP	City	Location type	Latitude	Longitude	Dwelling type	Building year	Floors	Ventilation type	Heating type	N. of inhabitants	Notes
1	67348	Obernai	Rural	48.464100	7.485700	House	1930	2	Natural	Oil	2	Two floors but the first one is the only used - elderly couple, non-smoker
2	67000	Strasbourg	Urban	48.572200	7.714400	Appartment	1930	1	Natural	Gas	3	Lots of traffic on the road, bus stop under the windows - Couple and a child, non-smokers
3	67350	Oberscha-effolsheim	Urban	48.587600	7.654200	House	1980	2	Mechanical	Gas	3	House on the edge of the fields, but in the city: more peri-urban than urban, but being the only dwelling in this case, choice to define as urban for the statistics - Couple and a child, non-smoker
4	67482	Strasbourg	Urban	48.590700	7.768100	Appartment	1970	1	Natural	Gas	1	Tenant, near parking - Single person, non-smoker
5	67000	Strasbourg	Urban	48.629000	7.732000	House	1980	NA	NA	NA	NA	NA
6	67172	Griesheim-prés-Molsheim	Rural	48.503600	7.524000	House	1980	3	Mechanical	Oil + wood	4	Bathroom air extraction, no more oil heating, old boiler room transformed into a bedroom, in the air corridor of Entzheim airport - Couple and two children, non-smokers
7	67000	Strasbourg	Urban	48.573000	7.792000	Appartment	1974	NA	NA	NA	NA	NA
8	67000	Strasbourg	Urban	48.331000	7.451100	House	1930	4	Natural	Gas	5	Residential area without too much traffic, significant treatment of the wood frame in the past (lindane?) - Couple and three children (including a teenager), non-smokers
9	67292	Mietesheim	Rural	48.878500	7.639300	Appartment	1920	4	Natural	Wood	6	Couple and four children (including two babies), smoking father

NA: Not Assigned,

Supplementary Material S2:

Standards of individual pesticides used:

2,4-D, 2,4-MCPA, acetochlor, alachlor, aldrin, allethrin, bifenthrin, buprofezin, chlorothalonil, clopyralid, cyphenothrin, cyprodinil, deltamethrin. dicamba, dichlobenil,dichlorprop, dieldrin, diflufenican, esbiothrin, flazasulfuron, fluroxypyr, imiprothrin, mecoprop-p,metazachlor, metolachlor, myclobutanil, o,p'-DDD, o,p'-DDE, o,p'-DDT, oxadiazon, p,p'-DDD, p,p'-DDE, p,p'-DDT, pentachlorophenol, permethrin, picloram, piperonyl butoxide, prallethrin, propachlor, propiconazole, tebuconazole, tetramethrin, triclopyr, trifluralin, α-cypermethrin, α-endosulfan, α-HCH, β-Endosulfan, γ-HCH, τ-fluvalinate

Supplementary Material S3: Retention time (RT), precursor ion and product ions with associated dissociation energy value in GC-MSMS for each pesticide.

	**Compounds**	**RT (mn)**	**Precursor ion (*m/z*)**	**Excitation voltage (V)**	**Product ions (*m/z*)**
**Internal Standards**	DDE d8	18.1	254	1.3.	184/149/219
	DDT d8	20.2	173	1.6	137/138
	nitrophenol d4	12.28	200	1.	139/154/182
	phthalate d4	24.43	153	1.	153
	trans-cypermethrin d6	27.54	183	0.9	168/165/153
	trifluralin d14	11.18	315	0.8.	267/209
**Pesticides**	2,4-D	14.3	213	1.5.	183/198/163
	2,4-MCPA	13.42	211	1.3.	183
	acetochlor	13.47	146	1.5.	131/118/91
	alachlor	13.64	160	0.9.	132/117/145
	aldrin	15	263	1.8.	193/191/227/228
	allethrin	15.36	123	1.2.	81/79/95/77/67
	bifenthrin	23.51	181	1.6.	166
	buprofezin	17.63	105	1.2.	77
	chlorothalonil	14.12	266	1.7.	170/205/231

	Compounds	RT (mn)	Precursor ion (*m*/*z*)	Excitation voltage (V)	Product ions (*m*/*z*)
	clopyralid	12.42	248	1.5.	204/168/146
	cyphenothrin	26.81	181	1.7.	152
	cyprodinil	15.63	224	2.1.	208
	deltamethrin	31.17	181	1.6.	152
	dicamba	13.14	279	1.4.	264/235/205
	dichlobenil	10.06	171	1.8.	100/136
	dichlorprop	13.7	245	1.2	209/229/217
	dieldrin	18.88	279	1.6.	243/241/206/209/207
	diflufenican	23	266	1.7.	218/245/183
	esbiothrin	15.36	123	1.2.	81/95
	flazasulfuron	12.49	231	1.5.	216/188/131
	fluroxypyr	17.15	253	1.5.	217/159/182
	imiprothrin	19.71	123	1.3.	81/95
	mecoprop-p	12.94	225	1.5.	209/197/163
	metazachlor	15.94	132	1.6.	117
	metolachlor	14.59	162	1.	133/118
	myclobutanil	18.9	179	1.2.	125/144/152
	o,p’-DDD	18.2	235	1.4.	199/165/200/163
	o,p’-DDE	16.7	246	1.9.	150/176
	o,p’-DDT	20.12	235	1.5.	199/165/200/163
	oxadiazon	17.29	175	1.1.	146/112/140
	p,p’-DDD	20.74	235	1.5.	165/163/199/200
	p,p’-DDE	18.07	246	1.9.	176/150
	p,p’-DDT	22.22	235	1.5.	165/199/163/200
	pentachlorophenol	16.86	323	1.5	288/214/252
	permethrin	27.33	183	1.5.	168/165/153
	picloram	19.19	297	2.	253/217/189
	piperonyl butoxide	22.05	176	1.4.	131/117/145/161
	prallethrin	15.82	123	1.3.	81/95
	propachlor	11.33	120	1.4.	117/103/92/77
	propiconazole	22.07	173	1.6.	144/108
	tebuconazole	23.42	125	1.4.	89/99

	Compounds	RT (mn)	Precursor ion (*m*/*z*)	Excitation voltage (V)	Product ions (*m*/*z*)
	tetramethrin	24.42	164	1.4.	107/93/135
	triclopyr	15	312	1.4.	254
	trifluralin	11.23	264	1.5.	206/188/171/160
	α-cypermethrin	28.96	181	1.8.	152
	α-endosulfan	17.81	241	1.6.	206/204/170/171
	α-HCH	12.26	183	1.3.	147/148/145/146/109
	β-Endosulfan	21.15	195	1.6.	159/160/123/125
	γ-HCH	12.83	183	1.4.	147/148/145/146/109
	τ-fluvalinate	29.91	250	1.7.	250

Supplementary Material S4: : ATD-GC/MSMS method performance and validation for pesticides analysis

Compounds	Equation	R^2	CV %	Recovery (%)	LOD (ng)	LOQ (ng)
dichlobenil	Y = 0.584311 × x + 0.0149806 × x^2	0.9998	25.7	95.5	0.25	0.08
trifluralin	Y = 0.0281514 × x + 0.00370642 × x^2	0.9971	27.5	81.6	0.01	0.002
propachlor	Y = −0.0193467 × x + 0.00101959 × x^2	0.9988	14.1	57.2	0.25	0.08
α-HCH	Y = 0.00314032 × x + 0.000537147 × x^2	0.9991	18.1	77.4	0.22	0.07
clopyralid	Y = −0.229071 × x + 0.00931519 × x^2	0.9988	4.7	59.8	0.25	0.08
flazasulfuron	Y = −0.2983 × x + 0.0353428 × x^2	0.9999	15.4	44.2	0.44	0.13
γ-HCH	Y = −0.664516 × x + 0.0304934 × x^2	0.9992	5.8	80.6	0.22	0.07
mecoprop-p	Y = 0.00562118 × x	0.9838	25.9	72.1	0.44	0.13
dicamba	Y = 0.0009972 × x + 6.67454e−005 × x^2	0.9986	10.4	54.8	0.44	0.13
2.4-MCPA	Y = 0.00172559 × x + 1.85696e−005 × x^2	0.9918	19.5	47.4	0.44	0.13
acetochlor	Y = 0.0116542 × x + 0.00024495 × x^2	0.9997	16.4	39.7	0.25	0.08

Compounds	Equation	R^2	CV %	Recovery (%)	LOD (ng)	LOQ (ng)
alachlor	$Y = -0.0646043 \times x + 0.0158739 \times x^2$	0.9999	20.0	80.9	0.44	0.13
dichlorprop	$Y = 0.253175 \times x + 0.00107106 \times x^2$	0.9921	29.2	47.2	0.44	0.13
chlorothalonil	$Y = 0.585031 \times x + 0.00132817 \times x^2$	0.9961	21.8	64	0.25	0.08
2.4-D	$Y = 0.139607 \times x + 0.00133056 \times x^2$	0.9976	22.3	40.3	0.44	0.13
metolachlor	$Y = 0.0450239 \times x + 0.000169866 \times x^2$	0.9995	17.7	46.9	0.44	0.13
aldrin	$Y = 0.0003578 \times x + 5.32758e{-}005 \times x^2$	0.9991	2.8	65.1	0.22	0.07
triclopyr	$Y = 0.0008511 \times x + 2.90409e{-}005 \times x^2$	0.9990	16.9	47.8	0.44	0.13
esbiothrin	$Y = -0.208025 \times x + 0.01518 \times x^2$	0.9929	10.2	62.5	0.22	0.07
allethrin	$Y = -0.0164852 \times x + 0.00809853 \times x^2$	0.9987	11.3	78.4	0.22	0.07
cyprodinil	$Y = 0.589763 \times x + 0.0100304 \times x^2$	0.9981	23.3	59.1	0.44	0.13
prallethrin	$Y = 0.0170286 \times x + 0.000584419 \times x^2$	0.9988	1.9	68.3	0.22	0.07
metazachlor	$Y = -0.0173222 \times x + 0.00198511 \times x^2$	0.9921	15.0	92	0.44	0.13
o.p'-DDE	$Y = 0.214587 \times x + 0.00369748 \times x^2$	0.9984	18.6	53.7	0.44	0.13
pentachlorophenol	$Y = 1.52246 \times x$	0.9982	6.8	47.2	0.44	0.13
oxadiazon	$Y = 0.259649 \times x + 0.0123259 \times x^2$	0.9988	6.4	62.8	0.44	0.13
fluroxypyr	$Y = 0.0219126 \times x + 0.000295729 \times x^2$	0.9988	14.2	62	0.25	0.08
α-endosulfan	$Y = -0.347037 \times x + 0.0550062 \times x^2$	0.9999	13.4	84.1	0.25	0.08
buprofezin	$Y = 3.1903 \times x + 0.00150316 \times x^2$	0.9980	1.7	87.4	0.44	0.13
p.p'-DDE	$Y = 0.3688745 \times x + 0.0256874 \times x^2$	0.9978	26.1	53.2	0.44	0.13
o.p'-DDD	$Y = 0.8796485 \times x + 0.00365894 \times x^2$	0.9968	15.7	38.5	0.44	0.13
myclobutanil	$Y = -0.0119564 \times x + 0.00126635 \times x^2$	0.9990	16.9	54.2	0.18	0.05
dieldrin	$Y = -0.0404649 \times x + 0.00861221 \times x^2$	0.9990	11.8	80.6	0.22	0.07
picloram	$Y = 0.02547874 \times x + 0.0014587 \times x^2$	0.9984	12.5	47.8	0.44	0.13

Compounds	Equation	R^2	CV %	Recovery (%)	LOD (ng)	LOQ (ng)
imiprothrin	$Y = 0.0129142 \times x + 0.00181641 \times x^2$	0.9984	10.1	69.5	0.22	0.07
p.p'-DDD	$Y = 2.356898 \times x + 0.0158745 \times x^2$	0.9990	4.1	54	0.44	0.13
o.p'-DDT	$Y = 1.254693 \times x + 0.3675841 \times x^2$	0.9999	11.3	53	0.44	0.13
β-endosulfan	$Y = 0.0507626 \times x + 0.000120145 \times x^2$	0.9992	8.0	72.1	0.22	0.07
propiconazole	$Y = 0.0189758 \times x + 0.874589 \times x^2$	0.9996	8.5	41.7	0.44	0.13
piperonyl butoxide	$Y = 0.281072 \times x + 0.00212207 \times x^2$	0.9857	17.1	32.8	0.44	0.13
p.p'-DDT	$Y = -8.06502 \times x + 0.356577 \times x^2$	0.9991	3.8	44.3	0.44	0.13
diflufenican	$Y = -0.01458741 \times x + 0.1547854 \times x^2$	0.9975	6.7	62.9	0.22	0.07
tebuconazole	$Y = 0.0291554 \times x + 0.000119102 \times x^2$	0.9999	33.5	65.1	0.44	0.13
bifenthrin	$Y = 0.282973 \times x + 0.0229783 \times x^2$	0.9999	18.4	67.7	0.22	0.07
tetramethrin	$Y = 0.0196582 \times x + 0.000315872 \times x^2$	0.9999	4.3	56.2	0.22	0.07
cyphenothrin	$Y = 6.14848 \times x + 0.0565313 \times x^2$	0.9999	5.5	74.5	0.22	0.07
permethrin	$Y = -0.00643462 \times x + 0.0177379 \times x^2$	0.9999	19.5	78.1	0.22	0.07
α-cypermethrin	$Y = 0.399647 \times x + 0.00944233 \times x^2$	0.9990	8.7	69.9	0.36	0.11
τ-fluvalinate	$Y = 0.445412 \times x + 0.00651781 \times x^2$	0.9964	6.9	45.2	0.36	0.11
deltamethrin	$Y = 0.315458 \times x + 0.0875697 \times x^2$	0.9996	25.0	42.2	0.44	0.13

Supplementary Material S5: Seasonal Variation of pesticides in air and dust samples, Asterisks (*) denotes denote means that are significantly different (P <0.05) between pyrethroids and non-pyrethroid pesticides in each matrix.

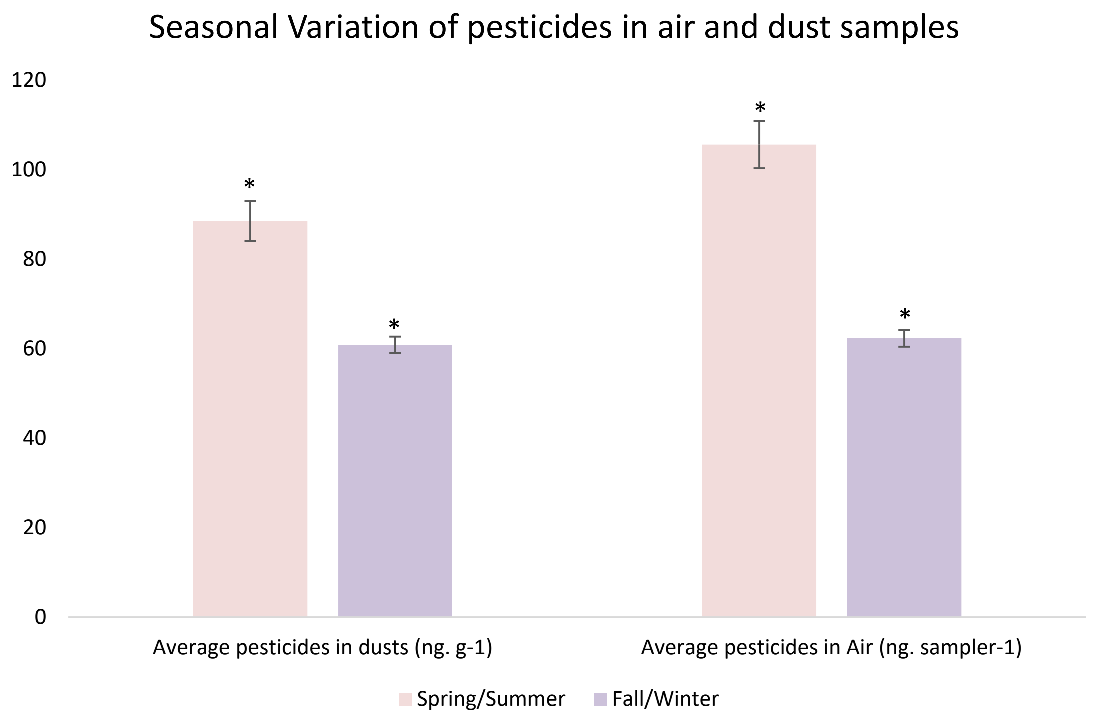

Supplementary Material S6: Classification of detected pesticides

Pesticide	Type
Propachlore	acetamide
Clopyralid	pyridine
γ -HCH	organochlorine
Chlorothalonil	dinitrile
Esbiothrine	pyrethroid
Cyprodinil	aminopyrimidine
Allethrin	pyrethroid
Prallethrin	pyrethroid
Buprofezine	thiadiazine
Imiprothrin	pyrethroid
Tebuconazole	triazole
Bifenthrin	pyrethroid
Cyphenothrin	pyrethroid
Permethrin	pyrethroid
α -cypermethrin	pyrethroid

** pesticides not belonging to pyrethroid are considered as "non-pyrethroids" pesticides.*

Reference	ZIP	City	Location type	Latitude	Longitude	Dwelling type	Building year	Last renoval	Floors	Ventilation type	Heating type	Number of inhabitant	Notes
1	67348	Obernai	Rural	48.464100	7.485700	House	1930	NA	2	Natural	Oil	2	Two floors but the first one is the only used - elderly couple, non-smoker
2	67000	Strasbourg	Urban	48.572200	7.714400	Appartment	1930	NA	1	Natural	Gas	3	Lots of traffic on the road, bus stop under the windows - Couple and a child, non-smokers
3	67350	Oberschaeffolsheim	Urban	48.587600	7.654200	House	1980	NA	2	Mechanical	Gas	3	House on the edge of the fields, but in the city: more peri-urban than urban, but being the only dwelling in this case, choice to define as urban for the statistics - Couple and a child, non-smoker
4	67482	Strasbourg	Urban	48.590700	7.768100	Appartment	1970	2012	1	Natural	Gas	1	Tenant, near parking - Single person, non-smoker
5	67000	Strasbourg	Urban	48.629000	7.732000	House	1980	NC	NC	NC	NC	NC	NC
6	67172	Griesheim-prés-Molsheim	Rural	48.503600	7.524000	House	1980	2005	3	Mechanical	Oil + wood	4	Bathroom air extraction, no more oil heating, old boiler room transformed into a bedroom, in the air corridor of Entzheim airport - Couple and

													two children, non-smokers
7	67000	Strasbourg	Urban	48.573000	7.792000	Appartment	1974	NC	NC	NC	NC	NC	NC
8	67000	Strasbourg	Urban	48.331000	7.451100	House	1930	NA	4	Natural	Gas	5	Residential area without too much traffic, significant treatment of the wood frame in the past (lindane?) - Couple and three children (including a teenager), non-smokers
9	67292	Mietesheim	Rural	48.878500	7.639300	Appartment	1920	NA	4	Natural	Wood	6	Couple and four children (including two babies), smoking father
